# Different religions, different animal ethics?

**DOI:** 10.1093/af/vfz047

**Published:** 2020-01-10

**Authors:** Louis Caruana SJ

**Affiliations:** Pontifical Gregorian University, Rome, Italy

**Keywords:** animal care, ethics, God, religion, virtue

**Implications**

- Religions, in spite of their differences, converge on some fundamental points, and some of these points concern our responsibility toward animals.
- Human superiority over all other creatures is to be understood in terms of caring for creation.
- Moral questions concerning our treatment of fellow humans are linked to those concerning our treatment of animals. Animal care is an obligation, both moral and religious.

## Introduction

Interest in animal ethics has recently increased considerably. This is due to various factors like technological progress, the sharp rise in human population, and the consequent pressure on global ecology. In this area, do traditional religions have anything to offer? It is obvious that religion still plays an important role in many areas of individual and communal life, for better or for worse. As regards animals, religious traditions affect the subliminal conscience and moral dispositions of billions of people. This paper explores this effect in three sections. The first section will be about religion, the second about conceptual clarification, and the third will be about morality.

At the very start however, an important general point needs to be highlighted. The paper’s title may give the impression that the overall argument will defend some form of relativism. The final result, however, will pull in the opposite direction. Accepting a plurality of perspectives is not the same thing as embracing relativism. The method adopted in this research acknowledges that, within the global, complex cultural landscape, each individual sees things from his or her own specific location. It acknowledges also however that being situated does not necessarily block the researcher from objective truth. Those who accept the relevance and importance of different cultural perspectives can still arrive at objective truths, just as observers can arrive at some truths about the room they are sitting in even though they are seated at different places.

## Religions and Animals

Starting with the most ancient traditions and proceeding chronologically, the following selective overview will first consider the main religions that emerged from India before spreading across East Asia: Hinduism, Buddhism, and Jainism; it will then deal with the Abrahamic religions, those that consider Abraham as their founder. In most religious traditions, animals play a symbolic role, but such symbolism will not be the focus of this paper. It will concentrate rather on moral issues, not limiting the discussion to animal-friendly teachings but mentioning also some problematic or negative aspects.

**Figure 1.**
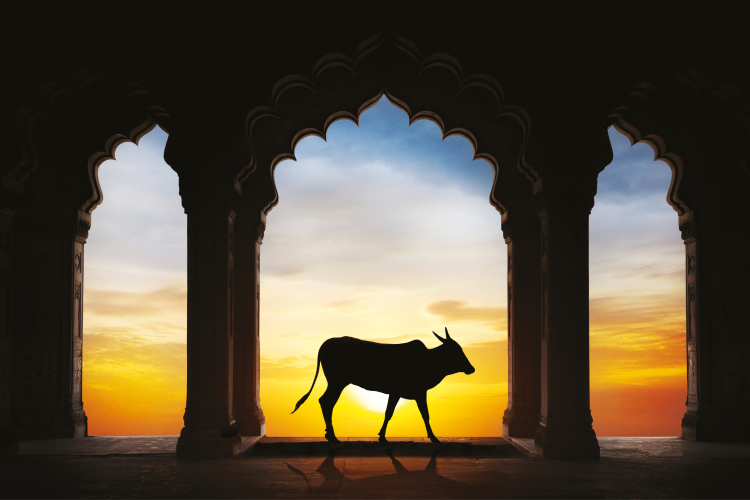
Holy Indian cow silhouette in old temple arch.

In Hinduism, the majority view as regards animals highlights two basic ideas: the idea of a hierarchy of living things with humans enjoying the highest status and the idea of reincarnation (Krishna, 2010; Kemmerer, 2012). The position of each animal within the hierarchy of life is not random, but determined by the fixed law of *karma*. Good deeds contribute to the believer’s promotion within the hierarchy, bad ones to a demotion. The idea of a hierarchy determines a kind of sacred inequality differentiating all biological species, differentiating even the various ethnic groups within humanity. This idea functions well within Hinduism for promoting good behavior, but it assumes that animals are situated at a significantly inferior level when compared with the lowest caste of humans. This devaluation of animals is counterbalanced by the many sacred texts, for instance in the Rig Veda and the Atharva Veda, where we find praise toward anyone who shows sensitivity toward animals. It is counterbalanced also by the belief that Hindu deities reincarnate as animals, especially as monkeys and cows, for instance Rama and Krishna. In fact, detailed studies indicate that the respect Indian religions show toward animals is supported by the strong symbolic link eventually established between the various animal species and the various divinities (Krishna, 2010). According to Nanditha Krishna, the cow veneration arose during the Vedic era. As is well-known, the cow occupies a special place in Hinduism, even today. In giving us milk, it represents our source: our mother or mother Earth. A relatively recent text, the Chandogya Upanishad, which appeared about 800 BC, confirms that nonviolence, or *ahimsa*, should be observed not only toward humans but also toward all beings (Figure 1).

As regards Buddhist traditions, one can start by highlighting a very general point. According to most interpretations, the goal of Buddhism is to overcome suffering and free oneself from the cycle of death and rebirth. One notices therefore that Buddhism retains from Hinduism the hierarchical view of beings and also the idea of reincarnation. It adds however the idea of personal liberation through enlightenment. The main goal for humanity is to find the right spiritual practice to end the suffering that results from rebirth. Later Buddhist interpretations hold that the painful cycle of rebirth occurs in six realms of existence: the heavenly, the demi-god, the human, the animal, the hungry ghost, and the hellish realm. The last three of these realms are evil, the animal realm included. Does Buddhism admit of a creator? This is a disputed question even today. One school holds that all phenomena originate from other phenomena and that the cycle of originating dependence is closed within itself. The universe therefore does not need a first cause. Other forms of Buddhism however admit the ultimate reality as the source of all things. For instance, Mahayana Buddhism describes the ultimate reality as the Womb of all Buddhas or as the Primordial Buddha. Regarding the status of animals, Buddhism shows trends that apparently pull in different directions. On the one hand, one maxim of the Noble Eightfold Path is that all Buddhists should refrain from killing. On a broad interpretation, this maxim includes all sentient life (Kemmerer, 2012). Consequently, vegetarianism is a highly respected ideal. On the other hand, Buddhism retains not only the hierarchy of life but also the idea that the animal realm is evil, in the sense that it is a realm that humans should avoid by living virtuous lives.

**Figure 2.**
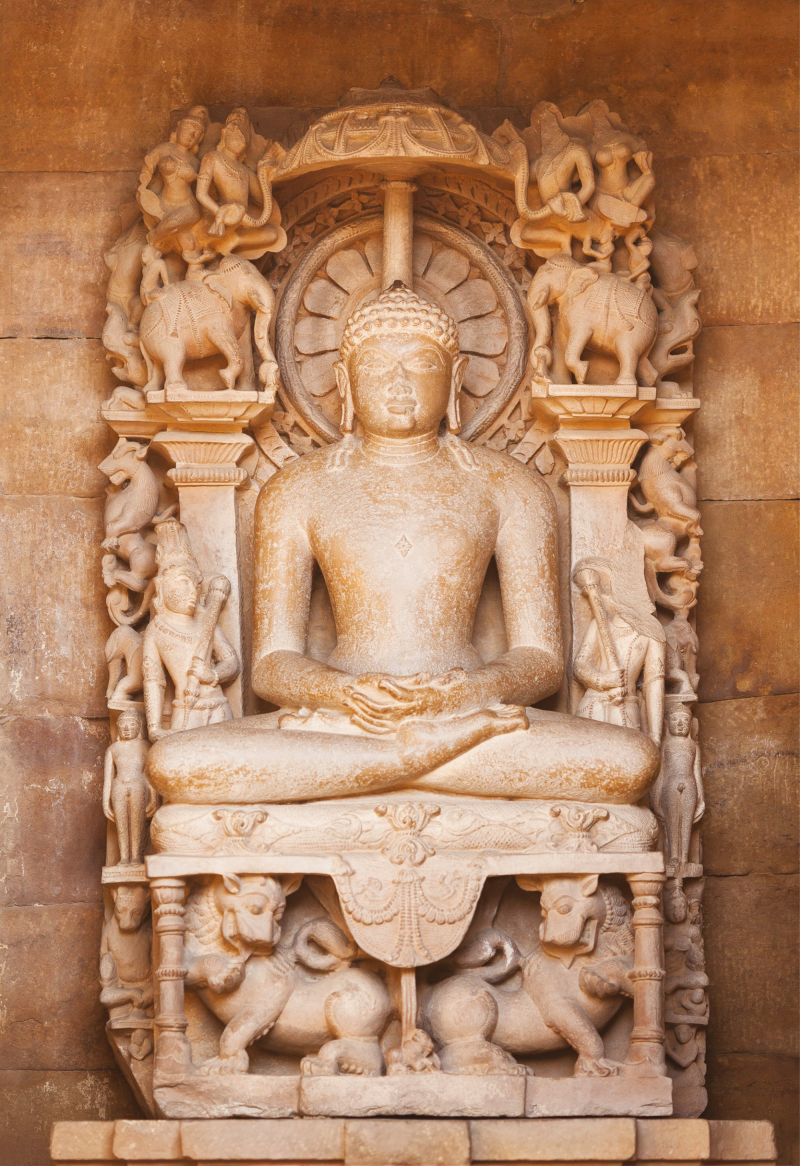
The Jain’s statue on a throne in an environment of elephants, lions, deities, and mythical animals in the Adinath Temple, Khajuraho.

Jainism is another ancient Indian religion. It is founded on the four main ideas of nonviolence, many-sidedness, nonattachment, and asceticism. Jain lifestyle is marked by vegetarianism and the avoidance of all harm to humans and animals. It is the strictest religion as regards avoiding harm to animals. All living things are meant to help one another. Killing is not allowed, even in self-defense. Going further than Hinduism and Buddhism, Jainism considers nonviolence the highest moral duty. The background cosmology is similar to what we saw in Hinduism and Buddhism, namely a hierarchy of living things and the cycle of rebirth, from which humans need to be liberated. According to some Jain traditions, killing is to be avoided not because of the inherent value of living things but to keep one’s soul pure, ensuring thus a better rebirth. One important prayer includes a plea for forgiveness from all living beings. The idea of *Jiva* corresponds somewhat to what Western thinkers call consciousness or soul but Jainism sees *Jiva* as present everywhere, in gods, humans, animals, plants, hell beings, and even in inert matter (Figure 2). There is, therefore, an emphasis on a common hidden vital principle that joins all things into a kind of brotherhood. The universe in all its realms is eternal and self-sufficient. There is no creator God who rewards and punishes. Instead, there is the law of *karma*. This plays the role of delivering reward and punishment and it does it through necessity.

**Figure 3.**
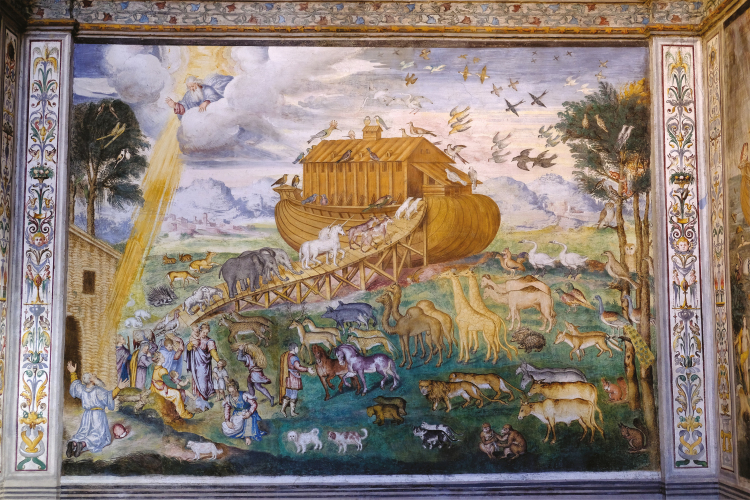
Noah, his family and two representatives of all the animals on the Earth enter the ark before the flood.

We move on now to the Abrahamic religions, starting with Jewish traditions. In the Jewish Bible, one finds that God created all things and that all creatures are good in themselves. There are also some specific moral obligations toward animals, for instance the injunction not to muzzle an ox while it is working (Deuteronomy 25:4), and to help a fallen overloaded donkey, even if it belongs to your enemy (Deuteronomy 22:4). The prophet Qohelet, speaking about the prospects after death, holds that “man has no superiority over beast” (Ecclesiastes 3:19 NRSV). More noteworthy still, one finds passages where the author describes animals as part of the human community. God commissions Noah to save not only his family but all creatures in view of a new world order (Figure 3). Moreover, after the flood, God establishes the new covenant with all creatures: “I am establishing my covenant with you [Noah] and your descendants after you, and with every living creature that is with you, the birds, the domestic animals, and every animal of the earth with you, as many as came out of the ark” (Genesis 9:9 NRSV). In the book of Jonah, the King’s call to fast, repent and return to living well, in line with God’s will, includes domestic animals (Jonah 3:7–9 NRSV). One could mention also human fellowship with animals as regards rest and as regards praise: “that your ox and your donkey may have rest” (Exodus 23:12 NRSV); “Let everything that breathes praise the Lord!” (Psalm 150 NRSV). The kosher slaughter of animals is allowed but it involves minimizing pain and draining away the blood to show respect toward the animal’s soul (Leviticus 17:10–13). Although a discussion on the related issue of animal sacrifice lies beyond the scope of this paper, one needs to mention at least one other somewhat disputed point. In the book of Genesis, there is an explicit reference to human authority and supremacy. “Then God said, ‘Let them [humans] have dominion over the fish of the sea, and over the birds of the air, and over the cattle, and over all the wild animals of the earth, and over every creeping thing that creeps upon the earth’” (Genesis 1:26 NRSV). According to many Jewish commentators, the idea here is that, since God is merciful toward all creation, humans should do likewise. They should imitate God by extending His mercy toward all creatures (Seidenberg, 2008; Kemmerer, 2012).

Christianity retained nearly all the religiosity of Judaism, articulated it to some extent in terms of Greek philosophy, and added its own original elements. As regards animals, the New Testament makes few direct references. Jesus did say of the birds that “not one of them is forgotten before God” (Luke 12:6 NRSV) but the main thrust of his message concerned humans. According to the Christian doctrine of the Incarnation, Jesus is both divine and human, and he invites humans to follow him and to become children of God. This idea entails a strong form of anthropocentrism. Nevertheless, it includes also a cosmological aspect. As explained by St. Paul, Christ’s salvific act embraces not just humans but all creation, including animals. Paul writes, “the creation itself will be set free from its bondage to decay and will obtain the freedom of the glory of the children of God. We know that the whole creation has been groaning in labor pains until now; and not only the creation, but we ourselves, who have the first fruits of the Spirit” (Romans 8:21–24 NRSV). Humans are definitely more important than animals. Nevertheless, many prominent Christian figures in history, like Francis of Assisi, became famous for their inclusion of animals as close friends, deserving love and mercy. For Catholics, official doctrinal statements focus not so much on whether animals have rights per se but on the moral constraints that apply to humans in their treatment of animals. The current position defends not only the unquestionable dignity of the human person but also the reality of moral obligations toward animals. On the one hand, the Second Vatican Council documents affirm that the human person is “the only creature on earth that God has willed for its own sake” (Paul VI, 1965, paragraph 24) and the Catechism of the Catholic Church (1994) adds that animals are “by nature destined for the common good of past, present, and future humanity” (Catechism, 1994, 2415). On the other hand, the same Catechism affirms that humans are obliged to “respect the particular goodness of every creature” (Catechism, 1994, 339). The recent encyclical *Laudato Sì* is more explicit. Pope Francis writes, “The ultimate purpose of other creatures is not to be found in us. Rather, all creatures are moving forward with us and through us towards a common point of arrival, which is God” (Francis, 2015, Section 83). Moreover, “our insistence that each human being is an image of God should not make us overlook the fact that each creature has its own purpose. None is superfluous” (Francis, 2015, Section 84). The overall current position emphasizes the urgent need for reconciliation with all creatures. Christianity is not a vegetarian religion. Nevertheless, it has always highlighted the importance of abstaining from the eating of flesh as a way to help realize the purity of life before the Fall, and thus prepare for the full realization of the new creation (Berkman, 2004).

The final point in this quick overview of major religions deals with Islamic traditions. Just like Judaism and Christianity, Islam recognizes God as Creator of a hierarchy of beings with humans on top. Humans enjoy a special status because they have a far higher dignity than animals. For Muslims, God created animals for the use of humans. For instance in The Qur’an (2004) Surah 16:5, there is the claim that “And livestock – He created them too. You derive warmth and other benefits from them: you get food from them.” Surah 40:79 says, “It is God who provides livestock for you, some for riding and some for your food.” Humans however are God’s vice-regents on Earth and are obliged to make decisions for the benefit of creation as a whole. Within Islam therefore, there is the same kind of anthropocentrism as in the other Abrahamic religions. Nevertheless, Muslims see animals as creatures that enjoy their own communities. Animals praise God in their own way, which we do not understand. For instance, the The Qur’an (2004) Surah 6:38 explains that “all the creatures that crawl on the earth and those that fly with their wings are communities like yourselves.” Later holy writings support these foundational ideas in The Qur’an (2004). Most significantly, the important Islamic collection, the Hadith, often describe the Prophet Muhammad’s special concern for animals. The central Islamic message of love, compassion, humility, submission, and almsgiving (*zakat*) is applicable not only for humans but also in the broader context of human-animal relations. The overall picture therefore has two sides. On the one hand, since humans are the centerpiece of creation, the killing of animals is permissible. On the other hand, maltreatment of animals is recognized as wrong. Killing for food therefore needs to be minimal and regulated carefully to minimize the painfulness of the procedure. The Qur’an (2004) in fact allows the eating of certain animals only, and only when slaughtered in a specified way.

## Conceptual Clarification

Each religion responds to the restlessness of the human heart by offering a particular viewpoint. Because of the various ramifications of religious traditions in the course of history, the overall stand as regards animals is not always clear. Nevertheless, we can still identify at least two areas of global convergence, one dealing with the interdependence between all living things and the other with the significance of the triad animality–humanity–divinity.

**Figure 4.**
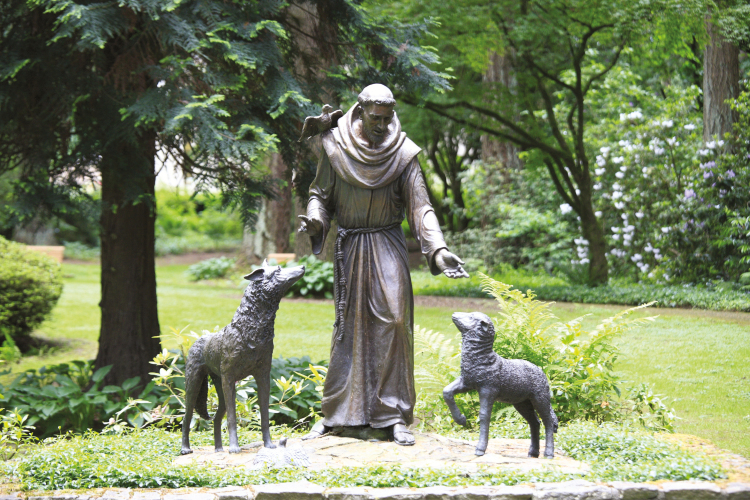
The Christian patron saint for animal care, St. Francis of Assisi.

First then: the interdependence of all creatures, material and spiritual. The very use of the word “creatures” reflects a common kinship. The universe, charged with its own dynamism, shows how most creatures flourish by using other creatures. Religions see therefore the entire biosphere as a unified, dynamic whole. This universal creaturely kinship is not a flat or chaotic landscape. It is a hierarchy. All living things occupy a specific position within this hierarchy. Humans may be the highest within the material realm but they are certainly not the highest overall. Our position bestows on us not only power and authority but also special responsibilities. The major religions accept that a lack of human respect toward animals often generates a corresponding lack of human respect toward other humans, especially the poor, the underprivileged, the physically or mentally challenged, the sick, and the old (Figure 4).

The second area of convergence involves the relation between the concepts of animality, humanity, and divinity. Religions go beyond the direct interest of animal ethicists, who normally focus on the animality–humanity relation. Religions add another dimension.

Many philosophers of ancient times, most notably Aristotle, had correctly recognized that humans are indeed animals, animals of a special kind. Nevertheless, our use of the term “animality” as distinct from “humanity” remains useful. Such use highlights the gap between us and other animals. “Animality” is sometimes used to refer to the bodily instincts of humans as distinct from human intellectual or spiritual nature. In what follows however, the focus will be mainly on animality as a generic characteristic of nonhuman animals. As regards animality in this sense, one notices first that it is not a human construct. Animality is a given. Although we can care for animals, manage them, dominate them, and eat them, we cannot construct them ourselves. Sometimes the expression “animal production” is indeed used, but this use is misleading. What we produce are things like tables and chairs. They are artifacts. Had humans never existed, the world would be bereft of tables and chairs. Not so as regards animals. They constitute part of the fundamental givenness of the world. Moreover, animality comes across to us as a realm of innocence. It is a morality-free zone. Sometimes, we might feel nostalgic about this zone. We might yearn for this state of life. We do share in animality but we are burdened, one might say, by another realm, the realm of thought and morality. Animality acts like a mirror that reveals something of our own nature to us. The gap is highly instructive (e.g., Derrida, 2002). It is certainly different from the gap between machinery and humanity. When we insert animals within complex input–output structures, designed for our benefit, we overlook the specific integrity that each animal represents. Factory farming degrades animality by confining it within the rigidity of machinery, within the restrictions of artificiality. In fact, in plain pragmatic and utilitarian terms, factory farming is nothing but the project “to raise as many animals as possible in the smallest possible space in order to maximize profits” (Degrazia, 1998, p. 281). The integrity of the individual animal does not count in any way. The problem here does not concern the factory only. It concerns the factory and all its links to society at large. The machine in this case includes its human administrators, its animal constituents and also the human consumers. The fact that consumers are far away, are ignorant of the conditions involved, or are unwilling to find out, does not detached them completely from the problem. By buying its products, consumers are in fact collaborating with the malpractice. The “social distance” between the perpetrator and the supporter of the system is never enough to render the supporter totally innocent. Some researchers therefore rightly support the demand for transparency and for boycotting. Current empirical studies have confirmed that many animals have rudimentary forms of beliefs, desires, and self-awareness (Degrazia, 1998; Lurz, 2009). Nevertheless, current levels of cruelty to animals are unacceptably high. For some people, awareness of this is like a personal wound, a wound that cannot heal. They carry it with them, hidden in their hearts, wherever they go, like a kind of original sin (e.g., Agamben, 2004; Cavell, 2009, p. 128–130).

As regards divinity, one needs to acknowledge that some religions, for instance Buddhism, apparently do not refer to God at all. Nevertheless, one can take divinity in a broad sense as a common element for all religions. Divinity in a broad sense refers to a transcendent order to which people aspire. The transcendent order is the ultimate goal and the source of moral insight. Religions talk about divinity in this sense in various ways, for instance in terms of union with a loving God or in terms of the dissolution of the self as a result of liberation from the cycle of rebirth. Whether Buddhism is fundamentally atheistic is a debated question and there is apparently no clear agreement between the various traditions. For instance, on the one hand, some argue that Buddhism is ultimately atheistic because of its deep conviction that the sense of unity between different aspects or experiences, as in our own subjective experiences, is an illusion. Therefore, things, although many, are not bound together by any kind of real unity (Hayes, 1988). On the other hand, in the Buddhist scriptures known as the Nibbana Sutta of the Udana Nikaya (the Pali Canon), one finds the Buddha himself teaching as follows: “there is, monks, an unborn-unbecome-unmade-unfabricated. If there were not that unborn-unbecome-unmade-unfabricated, there would not be the case that escape from the born-become-made-fabricated would be discerned. But precisely because there is an unborn-unbecome-unmade-unfabricated, escape from the born-become-made-fabricated is discerned” (Udana Nikaya, 2012). Such a statement indicates an ultimate One analogous to what the Abrahamic religions and various philosophies refer to. How does divinity, understood in this way, affect the animality–humanity conceptual relation? The divinity dimension opens up the horizon of religious believers to ideas about a common source and a common goal to all life. This horizon introduces a common ultimate relation of order and interdependence. Religious people feel obliged to care for animals, remaining nevertheless fully aware of their own human specificity of superior intellect and power. Are we ashamed of being so different from animals, so superior to them? The givenness of all life-forms includes the givenness of our own specificity. It includes our responsibility and the alarming ecological imperative that we are discovering nowadays, namely to care not just for ourselves but for all living things. This is a divine imperative, a commandment.

## Moral Implications

How does religion affect the foundational source of people’s action? Of course, actions speak louder than words. Religious doctrine therefore remains ineffective until it takes up concrete form in deliberation and action. Some personal traits or habits, attributes of the person as a whole, are crucial for that person’s morally good life. These traits are called virtues. Most religions and philosophical traditions agree that the basic virtues are not culturally dependent. They are the same for all people, whatever their culture or religion. Virtues like prudence, temperance, justice, and fortitude are universally indispensable for genuine human flourishing. How are these virtues applicable as regards animals? Let us consider them briefly one by one (Schaefer, 2008). In general, prudence makes one identify real needs and judge well as regards the best means to adopt. It ensures that one makes judgments in the light of all the available data. As regards animal welfare, this means that religious believers are motivated to collect all available data, including embarrassing data like appalling farming conditions and cruel slaughtering methods. Temperance, as sustained by religious discipline, helps believers avoid inordinate and immoderate desires, for instance excessive meat-consumption. Justice motivates religious believers to give to each his or her due, and to extend this imperative to all creatures. And finally fortitude: sustained by religion, this virtue makes believers act fearlessly even when opposed. With fortitude, they respond effectively to ecological concerns and are ready to revise well-entrenched practices. They are ready to engage in self-corrective procedures, even as regards their own belief systems, and to learn from past mistakes.

## Conclusion

This paper’s title was in the form of a question: “Different religions, different animal ethics?” Although most of the arguments presented deserve further exploration and analysis, the overall result is clear enough. There is considerable support for the claim that religions, in spite of their differences, do converge on some fundamental points; and some of these points regard animals. The conclusion can be formulated in two points. Firstly, a point about human superiority. The major religions indicate that it is indeed possible to affirm two apparently opposing claims: the claim that humans have a higher dignity than that of all other creatures and the apparently opposing claim that humans should not cause suffering to creatures. The way to hold these two affirmations together is to see human superiority in terms of caring for creation. Even though humans count more than animals, animals count as well. Indeed, they should count much more than what we have been assuming for centuries. Secondly, a point about urgency. One way of reacting to cruelty is to say that animals must wait. First, we need to learn how to eradicate cruelty to humans and then, once this is accomplished, we will sort out our relations with animals. This kind of response however is deceptive. We need to address all moral fronts together, in the right way. Practices like factory farming, irresponsible genetic manipulation, excessive meat-consumption, the use of animals for experiments, cosmetics or entertainment should all be thoroughly revised accordingly. Animal care is an obligation—both moral and religious.

## About the Author

**Louis Caruana** is Professor of Philosophy at the Pontifical Gregorian University, Dean of the Faculty of Philosophy and Adjunct Scholar at the Vatican Observatory. He started his education with a first degree in mathematics and physics and then proceeded with a master’s degree in philosophy (London) and a master’s degree in theology (Paris). After being ordained a Jesuit priest, he obtained his doctorate at the Department of History and Philosophy of Science, University of Cambridge. His pervious service includes teaching and research at Heythrop College, University of London, where he was appointed Reader. His research publications deal mainly with topics in philosophy of science, metaphysics, and philosophy of religion. He did extensive work on the concept of nature, modes of explanation, and Darwinism.
